# Lifecycle design of synthetic polymers: toward recyclability and sustainability

**DOI:** 10.1093/nsr/nwaf550

**Published:** 2025-12-02

**Authors:** Tao Xie, Yongfeng Men

**Affiliations:** State Key Laboratory of Chemical Engineering and Low Carbon Technology, College of Chemical and Biological Engineering, Zhejiang University, China; State Key Laboratory of Polymer Science and Technology, Changchun Institute of Applied Chemistry, Chinese Academy of Sciences, China

Synthetic polymeric materials are one of the greatest human inventions that have drastically changed our society. However, their excessive use and inappropriate end-of-life treatments have increasingly raised environmental concerns. In addition, discarded polymer products represent significant resource wastes. The increasing awareness of these issues has not only triggered policy changes, but also posed challenges for the scientific community to rethink synthetic polymers from the standpoint of life cycle design. This Special Topic is organized around these issues, covering various aspects including bio-based polymers, degradable polymers, and recyclable polymers.

Polyhydroxyalkanoates can be produced synthetically or by microorganisms. Focusing on the latter, Guo-Qiang Chen’s group reviewed recent progresses on this topic [1]. Special attention was paid on the biomanufacturing steps from environmental and energy consumption considerations. End-of-life options (biodegradation, anaerobic digestion, and chemical recycling) were discussed along with future challenges.

Poly(L-lactic acid) (PLLA) is a biodegradable bio-plastic, but its chemical recycling back into the monomer is challenging. This is because the process is accompanied by inevitable epimerization of L-lactide (L-LA) to meso-LA. Jian-Bo Zhu and collaborators addressed this issue with a catalyst that allowed stereoselective and sequence-controlled polymerization of L-LA in the presence of meso-LA [2]. The obtained stereogradient block copolymer showed uncompromised properties compared to the original PLLA. This epimerization-tolerant chemistry has the potential to drastically impact the future of PLLA. The mechanical performance of PLLA is a major hurdle for the widespread adoption of this sustainable polymer. Xiaoyan Tang and colleagues reported regioselective hydrogen-transfer polymerization of diacrylates followed by post-functionalization to yield polyester macroinitiators for synthesizing PLLA bottlebrush polymers [3]. These bottlebrush polymers, when used as toughening reagents, were found to drastically improve the ductility of PLLA.

Hu and Pang’s group reported the chemical design of backbone-editable polyesters and polycarbonates [4]. They illustrated that the flexibility to edit these polymers allowed adjusting of various polymer properties after their initial synthesis including hydroboration-oxidation reactivity, glass transition temperature, and thermal and hydrolytic degradation profiles. The latter, in particular, is highly relevant as tunable degradation provides the freedom to adjust the service life of the material to meet different demands.

For polyolefins, the superior stability of their carbon-carbon single bonds presents an enormous challenge for their chemical recycling. Introducing cleavable weak bonds into the backbones is a viable strategy to make polyolefins sustainable. Zhongbao Jian and colleagues reviewed advances on this subject including their synthesis, chemical modification, and recycling [5]. They outlined future challenges on designing polyolefins to achieve an appropriate structure-property-degradability balance.

The mixed nature of real-world plastic wastes make it challenging for their chemical recycling. Jinghong Li highlighted recent work from Ding Ma’s group on the chemical transformation of mixed plastics into useful products [6]. Guided by solid-state NMR analysis that identified the functional groups in mixed plastic wastes, a catalytic process was designed to transform them into potentially useful small molecular products. In a Perspective by Zhang and colleagues, pathways and challenges to deal with real-world mixed plastics were further discussed [7]. The authors outlined three possible pathways: mechanical recycling, non-selective decomposing plastic mixtures into basic chemicals, and selective conversion of plastics into monomers/oligomers or other high-value chemicals. They laid out key future considerations including economic viability, environmental impact, and scalability.

The stability of covalent bonds in commodity polymers creates inherent conflicts with their chemical recycling. Bo Qin’s group reported the depolymerization of both linear and cross-linked polyureas [8]. The underlying acylation-activation urea bond cleavage chemistry allowed regenerating the original diamine monomers that can be used for repolymerization. Most importantly, the simplicity and high efficiency of the process made it attractive both economically and environmentally.

Chemical recycling of thermoset polyurethane foams has been extensively investigated for decades, yet with limited success. Facing this challenge, Tao Xie’s group developed a simple one-step process that transformed the foam into a value-added 3D printing resin [9]. The process involved no catalyst/solvent, used bio-based reagents, and yielded 3D printed parts with superior mechanical properties.

One challenge of designing sustainable polymers is the trade-off between recyclability and in-use performance. Junqi Sun’s group addressed this issue by copolymerizing rigid poly(aryl amide) and flexible polyurea chains [10]. The polyurea phase formed nanodomains that reinforce the polymer with its hydrogen-bonding, which also serves as reversible crosslinking. The copolymer is not only reprocessable, but also exhibits a rare combination of high rigidity, strength, and toughness.

Closed-loop recycling represents an ideal solution for life-cycle design of synthetic polymers. Da-Hui Qu’s team reported an elegant example of an entropy-driven reversible ring-opening polymerization of polar-olefin-derived macrocycles catalyzed by an organic base [11]. By dissolution, the polymerization-depolymerization equilibrium was readily shifted to favor monomer recovery. The underlying principle represents a major departure from existing closed-loop recycling chemistry.

Achieving a good balance between monomer reactivity, polymer performance, and recyclability is important for developing recyclable polymers. A collaborative team between Xuzhou Yan, Shan Tang, and Bin Wang reported the ring-opening polymerization of a *cis*-fused cyclobutane-butyrolactone monomer [12]. The monomer ring strain contributed to high monomer reactivity as well as the high thermal stability of the polymer. The polyester was readily converted back into the monomer in the presence of tBuOK as the catalyst.

Sulfur-containing polymers are unique in their degradability, closed-loop recyclability, and high refractive index. Xuefeng Jiang’s group reviewed recent progress on this subject [13]. The review covered several aspects of this class of polymers, including synthetic methods, degradation mechanisms, and emerging applications. The authors suggested that future developments should move from the simple goal of replacing conventional plastics to multifunctional circular materials with great potential for use in multidisciplinary areas.

Sustainable development of synthetic polymers has gained considerable attention and is certain to impact the future of academic research as well as industrial evolution. The above papers highlight the importance of their life-cycle design. The question is: will there be a single closed-loop recyclable polymer that meets all future needs? The answer is no. This is because of the diverse requirements and restrictions from the user ends which are difficult to meet by one single polymer. For this reason, we see the need to continue developing diverse solutions in the future.
